# Prevalence and Associated Factors of Self-Medication among Pregnant Women on Antenatal Care Follow-Up at University of Gondar Comprehensive Specialized Hospital in Gondar, Northwest Ethiopia: A Cross-Sectional Study

**DOI:** 10.1155/2020/2936862

**Published:** 2020-09-29

**Authors:** Faisel Dula Sema, Deres Gezahegn Addis, Eshetie Azezew Melese, Demeke Dana Nassa, Zemene Demelash Kifle

**Affiliations:** ^1^Department of Clinical Pharmacy, School of Pharmacy, College of Medicine and Health Sciences, University of Gondar, Ethiopia; ^2^School of Pharmacy, College of Medicine and Health Sciences, University of Gondar, Ethiopia; ^3^Department of Pharmacology, School of Pharmacy, College of Medicine and Health Sciences, University of Gondar, Ethiopia

## Abstract

**Background:**

Self-medication is being prevalent throughout the globe. Although pregnant women are among the most vulnerable group of the population for drug-induced adverse effects on their fetus and themselves, many pregnant women use self-medication without adequate safety precautions.

**Objective:**

This study was aimed at assessing the prevalence and associated factors of self-medication among pregnant women on antenatal care follow-up at University of Gondar Comprehensive Specialized Hospital.

**Methods:**

A cross-sectional study was employed among 400 pregnant women attending antenatal care clinic at the University of Gondar Comprehensive Specialized Hospital between February 01 and May 30, 2019. A structured interviewer-administered questionnaire was used for data collection. Data were analyzed using SPSS® (IBM Corporation) version 22. Descriptive statistics were presented using frequency and proportion. Binary logistic regression was used to identify factors associated with self-medication with a 95% confidence level and *p* value of 0.05.

**Results:**

Among 400 respondents, the prevalence of self-medication during the current pregnancy was 44.8% (95% CI = 40.5-50). Among all respondents (400), 38.0% (95% CI = 33.3-42.8) and 12.5% (95% CI = 9.5-15) used herbal and conventional medicine, respectively. Self-medication showed a significant association with a previous history of self-medication and monthly income.

**Conclusions:**

The prevalence of self-medication among pregnant women is considerably high. The previous history of self-medication and monthly income showed a significant association with self-medication. Awareness creation should be done for reproductive-age women on the potential risks of self-medication.

## 1. Introduction

“Self-medication can be broadly defined as a decision made by a patient to consume a drug with or without the approval of a health professional” [1]. Traditionally, it has been defined as “the taking of drugs, herbs or home remedies on one's own initiative, or on the advice of another person, without consulting a doctor” [2]. “It involves the use of medicinal products by the consumer to treat self-recognized disorders or symptoms, or the intermittent or continued use of a medication prescribed by a physician for chronic or recurring diseases or symptoms. In practice, it also includes use of the medication of family members, especially where the treatment of children or the elderly is involved” [3]. Self-medication can be practiced by using both conventional/modern and herbal medicines (“Herbal medicine include herbs and/or herbal materials and/or herbal preparations and/or finished herbal products in a form suitable for administration to patients”) [4].

The prevalence of self-medication is alarmingly high throughout the globe. It ranges from 8.5 to 98%, showing variation with different subgroups of the population (e.g., male vs. female) and between a developed versus developing region [5].

Even though self-medication may have a considerable benefit as a part of self-care, it also poses many potential risks at both the individual and the community levels, as well as at the health care system [3]. It commonly associates with risks such as misdiagnosis, use of excessive drug dosage, prolonged duration of use, drug interactions, and polypharmacy [6]. Moreover, it is one of the major reasons for antibiotic resistance [7]. It may also cause skin problem, hypersensitivity, and allergy, especially in the case of those patients that do not follow the instructions given by the label of package insert [8].

Previous studies reported that the most frequent reasons for self-medications are having a minor illness, health care costs, lack of adequate time to visit a physician, prior experience in using a drug, and long waiting time to visit a qualified practitioner [5, 9]. In the developing world like sub-Saharan African countries where health care resource is very limited, people live with extreme poverty, and illiteracy rate is very high; the practice of self-medication could have a greater negative impacts, especially when practiced by very fragile population like pregnant women.

Since pregnant women are among the most vulnerable group of the population for drug-induced adverse effects on their fetus and themselves, the harmful effect of medication use is expected to be high if they are not used after prescription, supervision, and follow-up of authorized health care providers [10, 11], because the pharmacokinetic profile of the drug may be altered due to maternal physiologic changes during pregnancy. As a result, unexpected adverse drug reactions may occur. Moreover, medications may pass through the placenta and cause harm to the fetus [12].

Despite the occurrence of an adverse outcome that depends not only on the physical and chemical nature of the drug but also on the dose, duration, frequency, route of exposure, and gestational timing involved [11, 13], many pregnant women use self-medication without adequate safety precautions. The prevalence of self-medication among pregnant women was reported to be high throughout the world. It is estimated to be 22%-44% [14]. Furthermore, a relatively higher magnitude, up to 85%, is being reported from a resource-limited setting, particularly in Africa [15–20].

Pregnant women may use both over-the-counter and prescription medications like vitamins, analgesics, antibiotics, antimalaria, and herbal medicines for self-medication [15–22]. However, it is difficult to conclude that any drug is absolutely risk-free for pregnant women; even the most familiar and easily accessed drugs like nonsteroidal anti-inflammatory drugs are reported to have a risk of pregnancy adverse outcomes [23].

The low awareness of the potential risks of medication use during pregnancy [13, 24] and the relative difference in the knowledge, attitude, and sociocultural condition of pregnant women [19, 25–27] may cause the prevalence of self-medication in different settings to fluctuate. Unlike relatively resource-rich settings, self-medication may be highly widespread among illiterate, unemployed, women with low socioeconomic status, and women on their first trimester of pregnancy [18, 28–30].

In Ethiopia, the situation of self-medication among pregnant women could be worst due to limited access to health facilities, low socioeconomic status, relatively high illiteracy, and lack of awareness on the adverse effects of self-medication [9, 31–33]. Therefore, this study was aimed at assessing the prevalence and associated factors of self-medication among pregnant women on antenatal care follow-up at University of Gondar Comprehensive Specialized Hospital (UoGCSH), northwest Ethiopia. So, all possible necessary measures can be taken by a policymaker, health care providers, and all concerned stake holders of the area based on the findings of this study.

## 2. Methods

### 2.1. Study Design and Settings

An institution-based cross-sectional study was conducted among pregnant women attending the ANC (antenatal care) clinic at UoGCSH in Gondar town, northwest Ethiopia, from February 01 to May 30, 2019.

The study was conducted at UoGCSH, Gondar town, northwest Ethiopia, which is found at 738 km from Addis Ababa (capital city of Ethiopia). The hospital is the only referral hospital in the area of Gondar with multiple specialty clinics including an ANC follow-up clinic. It serves around 7 million people both rural and urban inhabitants. Currently, around 16,000 pregnant women visit the hospital for ANC services per year. At the ANC clinic, gynecologists, residents, interns, and midwife nurses participate in the delivery of ANC to pregnant women.

### 2.2. Population

All pregnant women who attended to the ANC clinic at UoGCSH were the source population. All pregnant women who were attending the antenatal care clinic at UoGCSH during the study period were the study population.

### 2.3. Inclusion and Exclusion Criteria

All pregnant women who visited the ANC clinic of the UoGCSH within the study period were included. Pregnant women who were critically ill, on labor pain, and unable to communicate at the time of data collection were excluded.

### 2.4. Sample Size Determination and Sampling Procedure

The sample size was determined by using a single-population proportion formula: *n* = (*Z*^2^(*P*)(1 − *P*))/*W*^2^, where *n* is the sample size required, *W* is the marginal error of 5% (*W* = 0.05), *Z* is the degree of accuracy required (95% level of significance = 1.96). *p* = 0.5 (50%) was used to increase the study precision by getting the maximum possible sample size. *n* = ((1.96)^2^(0.5)(1 − 0.5))/(0.05)^2^ = 384. With an addition of 5% nonresponse rate, the final sample size was 400.

By using simple random sampling, 400 patients were identified for data collection. According to the UoGCSH statistic and information office record, around 25 pregnant women visit the ANC clinic every day for ANC service. Thus, by calculating the number for the data collection period (60 days), we estimated the number of pregnant women (1500) who would visit the ANC clinic for ANC service. We have labeled 1500 lotteries, “label 1” for 400 pregnant women and “label 0” for 1100 pregnant women, and mixed them within a bag. Then, all pregnant women were asked to take out the lottery from the bag when they came for ANC service. Only pregnant women (400) who took out the lottery labeled as 1 were incorporated in the study.

### 2.5. Operational Definitions

Self-medication is the use of conventional/modern or herbal medicine by pregnant women without the prescription of authorized health care provider for the treatment of self-recognized disorders or symptoms or the intermittent or continued use of a medication prescribed by an authorized health care provider for chronic or recurring diseases or symptoms.

Illiterate is those who cannot read and write.

### 2.6. Data Collection Method and Procedures

Three clinical pharmacists were employed for the data collection. The data was collected by using a structured interviewer-administered questionnaire (Supplementary Materials Annex2). Although, the questionnaire was prepared in the English language after a thorough literature review of previously validated published studies [15, 16, 17, 18, 20–22], it was sent to one faculty member and two physicians to assess the face validity, because it was subject to slight modification. For internal consistency, we have used the alpha Cronbach test which had an index of 0.75. Then, the questionnaire was translated to the Amharic language by experts at the area and translated back to the English language to minimize translation error. It was pretested by 5% (20 pregnant women) of the sample size before the commencement of the actual data collection. The data collected for the purpose of the pretest was not included in the final analysis. Also, data was not recollected on individuals who had participated for the purpose of pretesting the questionnaire. The questionnaire has 33 questions categorized into 5 sections. The first section encompasses the sociodemographic information of the participants like age (in years), marital status, occupation, monthly income (in ETB), education level, religion, place of residence, and distance from health facility (hospital or health center). The second section, the obstetrics information of the participants, consists of the number of gravida, number of parity, number of children, history and reason of abortion, and stage of pregnancy. The third section contains pregnant women's attitude towards self-medication. In this part, pregnant women were asked whether they believe self-medication is important for maternal and fetal health and unusual health problem can occur on the infant(s) due to self-medication during pregnancy. They were also asked at which trimester of pregnancy the negative effect of self-medication can occur. The fourth and fifth sections comprise self-medication by conventional and herbal medicine, respectively. Under these parts, pregnant women were asked whether they have used conventional and/or herbal medicine during the current pregnancy for the purpose of self-medication. They were also asked for history of self-medication, reason for self-medication, and types of ailments treated by self-medication by both conventional and herbal medicines. The name, source of information, and source of medicine were asked for both the conventional and herbal medicines.

### 2.7. Data Quality Control

One-day training was given to the data collectors and supervisors on the objectives of the study, the contents of the questionnaire, and issues related to confidentiality before starting data collection. Data collection was supervised frequently. The questionnaire was pretested on 5% of the sample size.

### 2.8. Data Processing

The collected data were checked for completeness and consistency and then entered into SPSS® (IBM Corporation) version 22 for analysis. Also, the data was cleaned for possible errors. When there was an error, data were corrected by crosschecking with the data on the data abstraction format using the original ID variable on the SPSS immediately.

### 2.9. Data Analysis and Interpretation

At the end of data collection, data were coded, entered, and analyzed by using IBM SPSS version 22 software. Descriptive statistics were used to describe the result of the study in frequency, proportion, mean, and standard deviation. Independent variables were tested for association with self-medication (conventional and herbal medicine). Independent variables were also tested for association with conventional and herbal medicine separately. The association between dependent and independent variables was determined by using the chi-squared test (the results of the chi-squared test is submitted separately as Supplementary Materials Supporting information) and binary logistic regression. The chi-squared test was used to apply Fisher's exact test for identifying variables which could not fulfill the assumption for the Pearson chi-squared test and logistic regression. The score was dichotomized as yes = pregnant women with self-medication practice and no = pregnant women with no self-medication practice. The Pearson chi-squared test and Fisher exact test of independence were used to determine the association between the independent and dependent variables. Then, we have checked the magnitude of the association by using binary logistic regression for those variables that fulfill the assumption for model fitting. Statistical significance was considered when a *p* value was less than 0.05. Although we had perform analysis by both chi-squared test and binary logistic regression, we preferred to report our finding from the binary logistic regression, since it is a more stronger analysis method. The independent variables were checked for multicollinearity (with variance inflation factors for all models 1 = 1.079) and the presence of outliers (with a probability of Mahalanobis distances = 0.06). The bivariable logistic regression analysis was done to identify an association between the dependent and each independent variable. The model was tested by using Hosmer–Lemeshow goodness of fit (model 1: chi-squared test = 1.615, df = 7, sig. = 0.978; model 2: chi-squared test = 18.209, df = 7, sig. = 0.011; and model 3: chi-squared test = 5.397, df = 7, sig. = 0.612). All independent variables with *p* < 0.2 in the bivariable logistic regression analysis were entered for multivariable analysis using the enter method. Finally, the result of the study was presented in tables and pie chart.

### 2.10. Ethical Consideration

To conduct the study, ethical clearance and approval were obtained from the Ethical Review Committee Department of School of Pharmacy, College of Medicine and Health Sciences, and then, formal permission was obtained from the medical director of UoGCSH. Then, written informed consent (Supplementary Materials AnnexI) was obtained from the study participants after the objective of the study was made clear and permission for cooperation was asked politely.

## 3. Results

### 3.1. Sociodemographic Information of the Respondents

A total of 400 pregnant women participated in this study with a response rate of 100%. From the entire participant, 199 (49.8%) women were at the age of 28–37 years, with a mean and standard deviation of 27.83 ± 4.27 years. Most of them (*N* = 364, 91.0%) were married. Concerning their occupation, 126 (31.5%) women were a housewife. According to the WHO income scale level, 159 (39.8%) women had a low monthly income (<3000 ETB (Ethiopian birr)). Less than half of the pregnant women had a diploma/degree (*N* = 162, 40.5%). The commonest religion of the participant was orthodox (*N* = 305, 76.3%). For most pregnant women, place of residence was urban (*N* = 337, 84.3%) with a distance from health facility less than 5 km (*N* = 237, 59.3%) (Table 1).

### 3.2. Obstetrics Information of the Respondents

One hundred fifty (67.5%) pregnant women were gravida II. Most of the respondents (*N* = 159, 39.8%) had one child with parity one. The previous history of abortion was recorded on 26 (6.5%) pregnant women. The major reason for their abortion was a health problem (*N* = 14, 3.5%). Most of the respondents (*N* = 300, 75%) were in the third trimester of pregnancy (Table 2).

### 3.3. Pregnant Women's Attitude towards Self-Medication

Among 400 pregnant women, 35 (8.8%) of them believed that self-medication is important for maternal health and 8 (2.0%) of them believed that self-medication is important for the health of the unborn child. Around one-third of the pregnant women thought that the negative effect of self-medication occurs during the first trimester (*N* = 261, 65.3%). Also, 289 (72.3%) of 400 pregnant women had a belief that unusual health problem can occur on the infant(s) due to self-medication use during pregnancy (Table 3).

### 3.4. Prevalence of Self-Medication

**Figure 1:**
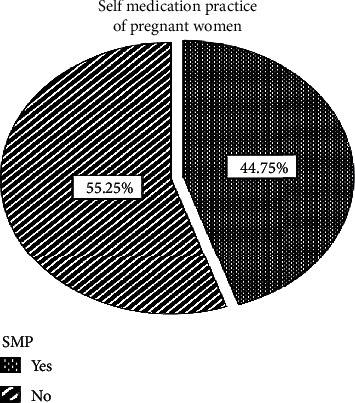
Self-medication of pregnant women at UoGCSH, June 2019.

Among 400 pregnant women, 179 (44.8%) (95% CI = 40.5-50) of them used self-medication during the current pregnancy (Figure 1). It includes the prevalence of both conventional 50 (12.5%) (95% CI = 9.5-15) and HM 152 (38.0%) (95% CI = 33.3-42.8) (Tables 4 and 5). Around one-third of the participants, 146 (36.5%), had a previous history of self-medication (Table 4).

### 3.5. Self-Medication with Conventional Medication

In this study, around half of the CM users (*N* = 26, 52.0%) had prior experience of the drug. Headache was the main ailment sated for CM use (*N* = 30, 60%). The commonly used drug was paracetamol (*N* = 29, 58.0%). Pharmacists/druggists were the most used source of information about the drug (*N* = 37, 74.0%). Private community pharmacies/drug stores (*N* = 46, 92.0%) were the major sources of the drugs. However, most of the users have no information on the dose and side effects of the drugs they used (*N* = 28, 56.0%). Around 149 (37.3%) of the study participants had previous history of self-medication by conventional medicine (Table 4).

### 3.6. Self-Medication with Herbal Medicine

The study showed that 152 (38.0%) pregnant women used HM. The most frequent disease for HM use was common cold (*N* = 69, 44.08%). Ginger was a commonly used HM (*N* = 87, 55.92%). The users mainly got information about HM from neighbors (*N* = 77, 48.68%). In most cases, pregnant women prepare HM by themselves (*N* = 146, 94.73%). However, 107 (70.39%) of them could not state the specific reason for HM use for self-medication. Half of the participants, 200 (50%) of them, had previous history of self-medication by herbal medicine (Table 5).

### 3.7. Factors Associated with Self-Medication

The only variables which fulfill the assumption for model fitting were age, monthly income, and previous history of self-medication. Pregnant women having previous history of self-medication associated with self-medication 26.816 (13.064, 15.064; *p* value = 0.001) times more likely than pregnant women with no previous history of self-medication. Pregnant women who had monthly income greater than 6000 Ethiopian birr associated 2.441 (1.197, 4.977) times more likely than pregnant women with monthly income less than 3000 Ethiopian birr with an overall *p* value of 0.022 (Table 6).

### 3.8. Factors Associated with Self-Medication with Conventional Medication

By binary logistic regression, the only variable associated with self-medication with CM was previous history of self-medication. Pregnant women who had previous history of self-medication associated with self-medication with CM 5.223 (2.007, 13.592; *p* value = 0.001) times more likely than pregnant women with no previous history of self-medication (Table 6).

### 3.9. Factors Associated with Self-Medication with Herbal Medicine

The previous history of self-medication and monthly income significantly associated with self-medication with HM. The previous history of self-medication associated with self-medication of pregnant women with HM around 27.164 (11.913, 61.935; *p* value = 0.001) times more likely than pregnant women with no previous history of self-medication. Similarly, pregnant women with monthly income greater than 6000 Ethiopian birr associated with self-medication with HM 2.739 (1.376, 5.452; *p* value = 0.004) times more likely than pregnant women with monthly income of less than 3000 Ethiopian birr with overall *p* value = 0.005 (Table 6).

## 4. Discussion

This study assessed the prevalence and associated factors of self-medication among pregnant women who were attending to the ANC clinic of UoGCSH. In this study, we have reported the prevalence and associated factors of self-medication as a whole to have the overall picture of it. In addition, we are also convinced to report the prevalence, reasons, ailments, type of medicine, source of information, source of the medicine, and associated factors for both conventional and herbal medicines separately, because some of the previous studies reported self-medication by considering only either conventional or herbal medicine.

The prevalence of self-medication during the current pregnancy was high, 44.8% (95% CI = 40.5-50). This is a composite of both conventional and herbal medicine use. This finding is consistent with the study done in Mwanza and Tanzania (46.24%) [29]. However, it is higher than the studies done in Addis Ababa, Ethiopia (26.6%) [20], Goba town, southeast Ethiopia (15.5%) [21], Iran (35%) [25], and Mexico (21.9%) [34]. On the contrary, it is lower than the study done in Harar town, Ethiopia (69%) [16]; Nigeria (85%) [18]; Bukavu in Eastern DR Congo (61.3%) [17]; and Central Region of Ghana (69%) [35]. When we look into its subparts, conventional and herbal medicines, the prevalence of CM practice was 12.5% (95% CI = 9.5-15). It is higher than the study done in Mexico (6.11%) [34]. Conversely, it is lower than the studies done in Harar town, Ethiopia (29.1%) [16], and Addis Ababa, Ethiopia (18.2%) [20]. The prevalence of HM practice was 38.0% (95% CI = 33.3-42.8). It is higher than the study done in Addis Ababa, Ethiopia (10.9%) [20]; Mexico (14.68%) [34], Gulu district in Northern Uganda (20%) [22]; and Mwanza in Tanzania (25.3%) [29]. In opposite, it is lower than the study done in Gondar, Ethiopia (48.6%) [15], and Harar town, Ethiopia (58.2%) [16]. The difference may be due to the diversity in the socioeconomic factors, geographic location, accessibility, and study settings.

The high prevalence of self-medication by pregnant women may be due to multiple reasons. From these, the lack of knowledge of pregnant women on the potential risks of self-medication during pregnancy could be among the major one. Like most of sub-Saharan African countries, because of the high work load on the health care workers and patient flow on the study setting, health care providers may not give adequate health education on the possible risks of self-medication on the pregnant women's health and their fetus adequately. In addition, the attitude of the pregnant women towards self-medication can have a significant impact on the prevalence. In this study, we have noticed that 35 (8.8%) of the pregnant women believed that self-medication is important for maternal health and 8 (2.0%) of them believed that self-medication is important for the health of the unborn child. Moreover, 21% of them had no idea whether self-medication during pregnancy is important or harmful for fetal health. Similarly, 30.5% of them had no idea at which trimester the negative effect of self-medication occurs and 24.5% of them also had no idea on whether unusual health problem can occur on the infant(s) due to self-medication use during pregnancy (Table 3). The awareness creation of pregnant mothers about self-medication to improve their knowledge and attitude of potentially childbearing aged women particularly pregnant women towards self-medication through different techniques like mass-media and inclusion of the issues of self-medication as a routine counseling point during ANC clinic follow-up may have a reasonable contribution to counteract the challenge.

However, despite having a good knowledge and attitude of self-medication, pregnant women may practice self-medication for other various reasons including sociocultural and economic reasons [16–18, 20, 21, 35, 36]. Thus, the pregnant women were asked the reasons for self-medication by both conventional and herbal medicines. The commonest reasons given for the conventional and herbal medicine use were having prior experience of the drug (52%) and easy accessibility (23.6%), respectively. This is supported by the study done in Harar town, Ethiopia [16]; Addis Ababa, Ethiopia [20]; Bukavu, Eastern DR Congo [17]; and Jos, Nigeria [18]. Additionally, knowing about the disease and treatment was the reason for self-medication by CM. Likewise, the less frequent reasons for HM use were better effectiveness, lower cost, and fewer side effects than CM (Harar town, Ethiopia) [16]. The other commonly mentioned reasons for self-medication in previous studies were time-saving [20, 21], the disease was not serious (61) (54.5) [20, 35, 36], lack of trust in drugs prescribed by health workers [21], cheaper treatment cost, positive outcomes [35], expensive drug prescription in the health facility, and availability of old prescription [36]. Even though pregnant women self-medicate due to prior experience of the medicine and knowing about the disease and treatment, it does not mean the medicine is free from any potential risk for the fetus. The risk may be overwhelming especially for herbal medicines in countries like Ethiopia where there is no standardization of the dose, frequency, and duration of herbal medicine. So, due to the various nature of the reason given for self-medication, interventions should be based on case-by-case scenario. In addition to well-planned awareness creation, improving health care accessibility, strengthening the legal restriction of the dispensing of medicine without prescription for pregnant women, and financial empowerment of pregnant women may be helpful.

Paracetamol (58.0%) and ginger (55.9%) were the most commonly used conventional and herbal medicines, respectively. This is consistent with many previous studies [15–17, 20, 21, 26, 34]. Due to the easy accessibility of paracetamol as over-the-counter (OTC) medicine and ginger on the free market, many pregnant women were motivated to self-medicate with these agents. Even though these medicines are reported to be relatively safe, there are also evidences on potential risk of paracetamol and ginger on the pregnancy adverse outcomes if they are not used under precaution [37–39]. In addition, due to lack of standardization of traditionally used medicine and inability of most pregnant women to understand the package insert prepared in the English language, many self-medicating women may use the medicines for the wrong indication, dose, and duration, which may result in adverse pregnancy outcome.

Headache (60.0%) and common cold (44.08%) were the most commonly mentioned ailments for the use of conventional and herbal medicine, respectively. In addition, self-medication was practiced for treating nausea/vomiting, typhoid, and cough. It is in line with many of the previous studies [15–18, 29, 34]. Because of physiologic disturbance during pregnancy, pregnant women may be prone to many illnesses; however, pregnant women should consult their doctors before taking any medication to minimize the potential risks of self-medication.

Pharmacist/druggist (74.0) for CM use and neighbors (48.68) followed by family and friends (46.05) for HM were the commonest source of information about self-medication. This is consistent with studies done in Harar town, Ethiopia [16]; Gondar, Ethiopia [15]; Mexico [34]; and Addis Ababa, Ethiopia) [20]. So, empowering pharmacists/druggists, consideration of families, close relatives, and friends in the health education of pregnant women may prevent potential risk and maximize the possible benefit of self-medication.

The conventional medicines used were mostly obtained from a community pharmacy or drug store (92.0). However, the HMs were mainly self-prepared (94.7%). It is consistent with the previous studies done in Harar town, Ethiopia [16], and Addis Ababa, Ethiopia [20]. However, HMs were purchased from the supermarket in the study done in Mexico [34]. This may be due to the presence of tradition of herbal remedy preparation at home in Ethiopia. However, most of the respondents (56.0%) in this study did not have adequate information about the drug. So the preparation of local standard guidelines and availability to the population for herbal medicine and capacity building of pharmacy professionals regarding commonly used medication by pregnant women, especially OTC medication, may prevent risks and encourage more safe self-medication.

In this study, self-medication significantly associated with a previous history of self-medication and monthly income. However, in the study done in Hosanna town, southern Ethiopia [28], it strongly associated with pregnant women who were unable to read and write followed by women who had health problems during pregnancy and those who can read and write and had primary education [28]. In the study done in Tanzania, it showed a significant association with illiteracy, being unemployed, and being in the first trimester of pregnancy [29]. In the study done in Mexico [34], it significantly associated with smoking followed by alcohol consumption and higher education [34]. The discrepancy in the predictors of self-medication in different studies may be due to difference in study setting. So, it is a good reflection that studies are very essential to identify the driving factors and better understand the nature of self-medication in different settings. In addition, since some of the previous studies only consider either conventional or herbal medicine as self-medication, it may be appropriate to identify factors for each of them independently.

In this study, CM use significantly associated with a previous history of self-medication. This may be due to the sense of perceived experience to the CM. Thus, pregnant women may believe that using drugs that were used out of pregnancy may not harm their pregnancy. The remaining medications that had been obtained before the current pregnancy at home may be used for the same symptoms or illnesses that they had had before they were getting pregnant. However, it could be a harmful practice because previously used medicines may not be safe for pregnant mothers.

Similarly, the use of HM significantly associated with previous history of self-medication and monthly income. Likewise, herbal medicine was highly practiced by pregnant women with relatively higher monthly income (greater than 6000 Ethiopian birr). This may be due to affordability issue of locally available herbal medicine. However, lower monthly income (less than 3000 Ethiopian birr) was tending to associate with conventional medicine. It seems obvious that pregnant women with lower monthly income tend to practice self-medication by conventional medicine, because they may not afford the necessary health care cost to visit the nearby health care facility.

Considering the high prevalence of self-medication and variability in the magnitude, reasons, the types of illness, and medicines used for self-medication, all concerned bodies should pay due attention to the self-medication of pregnant women. Health care providers may contribute a lot through education, provision of adequate information, and counseling of pregnant women about self-medication depending on case-by-case circumstances.

## 5. Limitations

A recall bias among the participants and being shy to tell the truth because the study was institutional and the data were collected through interviewer-administered structured questionnaire may reduce the magnitude of self-medication.

## 6. Conclusions

The reported prevalence of self-medication among pregnant women is considerably high. Pregnant women use both herbal and conventional medicines for the purpose of self-medication.

However, a relatively higher prevalence of herbal than conventional medicine use is reported by pregnant women. Previous history of self-medication significantly associates with self-medication. It also significantly associates with herbal and conventional medicine use independently. Monthly income showed a significant association with self-medication in general and self-medication with herbal medicine.

Given that self-medication is common, awareness creation should be done for reproductive-age women about the risks of self-medication by both herbal and conventional medicines through different techniques. In addition, health care providers, especially those who are involved in ANC, should be aware of evidence regarding the prevalence, potential benefits, and harms of self-medication. A detailed study on self-medication is also recommended, particularly to establish the efficacy, safety, and standardization of commonly used herbs during pregnancy.

## Figures and Tables

**Table 1:** Sociodemographic information of the respondents at UoGCSH, June 2019.

Characteristics		*N* (400)	%
Age group in years	18-27	193	48.2
	28-37	199	49.8
	>38	8	2.0

Marital status	Single	12	3.0
	Married	364	91.0
	Divorced	40	5.0
	Widowed	4	1.0

Occupation	Governmental employed	125	31.3
	Self-employee	106	26.5
	Housewife	126	31.5
	Farmer	23	5.8
	Student	9	2.3
	Unemployed	11	2.8

Monthly income in ETB^∗^	<3000	159	39.8
	3000-6000	135	33.8
	>6000	106	26.5

Education level	Illiterate	23	5.8
	Primary school [1–8]	57	14.3
	Secondary school [9–12]	151	37.8
	College/university student	7	1.8
	Diploma/degree	162	40.5

Religion	Orthodox	305	76.3
	Muslim	80	20.0
	Protestant	14	3.5
	Jehovah witness	1	0.3

Place of residence	Urban	337	84.3
	Rural	63	15.8

Distance from a health facility	<5 km	237	59.3
	5-10 km	102	25.5
	>10 km	61	15.3

^∗^ETB: Ethiopian birr.

**Table 2:** Obstetrics information of the respondents at UoGCSH, June 2019.

Characteristics		*N* (400)	%
Number of gravidae	One	120	30.0
	Two	150	37.5
	Three	93	23.3
	≥Four	37	9.4

Number of parity	No child	125	31.3
	One child	159	39.8
	Two children	84	21.0
	More than two children	32	8.2

Previous abortion	Yes	26	6.5
	No	374	93.5

Reason for abortion (*N* = 26)	Health problem	14	3.5
	Low economic level	1	0.3
	Unwanted pregnancy	5	1.3
	Unspecified	6	1.5

Stage of pregnancy	First trimester	11	2.8
	Second trimester	89	22.3
	Third trimester	300	75.0

**Table 3:** Pregnant women's attitude towards self-medication at UoGCSH, June 2019.

Characteristics		*N* (400)	%
Self-medication is important for maternal health	Yes	35	8.8
	No	365	91.3

Self-medication is important for fetal health	Yes	8	2.0
	No	308	77.0
	I have no idea	84	21.0

I believe that the negative effect of self-medication occurs at the X^a^ week of pregnancy	First trimester	261	65.3
	Second trimester	13	3.3
	Third trimester	4	1.0
	I have no idea	122	30.5

I believe that unusual health problem can occur on the infant(s) due to self-medication use during pregnancy	Yes	289	72.3
	No	13	3.3
	I have no idea	98	24.5

^a^X represents first trimester, second trimester, third trimester, and I have no idea.

**Table 4:** Self-medication among pregnant women by conventional medicine at UoGCSH, June 2019.

Characteristics		*N*	%
CM use during the current pregnancy(*N* = 400)	Yes	50	12.5
	No	350	87.5

Reasons for CM (*N* = 50)	Easily available	8	16.0
	Better knowledge about the disease and the treatment	5	10.0
	Had prior experience on the drug	26	52.0
	Unable to specify the reason	11	22.0

Ailments for CM (*N* = 50)	Headache	30	60.0
	Nausea/vomiting	3	6.0
	Typhoid	4	8.0
	Common cold	4	8.0
	Cough	8	16.0
	Unspecified	1	2.0

Drugs for CM (*N* = 50)	Paracetamol	29	58.0
	Amoxicillin	8	16.0
	Cough syrup	2	4.0
	Hyoscine	2	4.0
	Metronidazole	4	8.0
	I do not know the name of the drug	5	10.0

Source of information about the drugs (*N* = 50)	From friends	4	8.0
	Internet	3	6.0
	Pharmacist/druggist	37	74.0
	Other health professionals	6	12.0

Where did you get the drugs (*N* = 50)	Neighbors	4	8.0
	Private community pharmacies/drug stores	46	92.0

What did you know about the drugs (*N* = 50)	Dose	14	28.0
	Side effect	8	16.0
	No information	28	56.0

Previous history of self-medication by conventional medicine	Yes	149	37.3
	No	251	62.7

^∗^Some of the pregnant women use both conventional and herbal medications.

**Table 5:** Self-medication among pregnant women by using herbal medicine at UoGCSH, June 2019.

Characteristics		*N* = 400	%
HM during the current pregnancy	Yes	152	38.0
	No	248	62.0

Reason for HM use (*N* = 152)	Herbal medicines are effective than conventional medicines	5	3.28
	Herbal medicines have fewer side effects	3	1.97
	Herbal medicines have a lower cost	1	0.66
	Herbal medicines are accessible without a prescription	36	23.68
	Unable to specify	107	70.39

Ailments (*N* = 152)	Headache	65	42.76
	Nausea/vomiting	17	11.18
	Typhoid	1	0.66
	Common cold	67	44.08
	Unable to specify	2	1.32

Types of herb(s) (*N* = 152)	Ginger	85	55.92
	Garlic	2	1.31
	Ocimum lamiifolium	60	39.45
	Unable to specify	5	3.28

Source of information about HM (*N* = 152)	Traditional healers	2	1.32
	Health professionals	6	3.95
	Family and friends	70	46.05
	Neighbors	74	48.68

Where did you get the herbal medicine? (*N* = 152)	Self-preparation	144	94.73
	Traditional healers/herbalist	1	0.66
	Traditional birth attendants	1	0.66
	Neighbors	8	3.95

Previous history of self-medication by herbal medicine	Yes	200	50
	No	200	50

**Table:** (a) Model 1: binary logistic regression analysis of factors associated with self-medication

Characteristics		Self-medication		COR with 95% CI	AOR with 95% CI	*p* value
		Yes	No			
Age group in years	18-27	74	119	0.604 (0.406, 0.899)	0.776 (0.474, 1.274)	0.315
	>28	105	102	1.655 (1.112, 2.465)		

Monthly income						0.022
	<3000	72	87	1	1	
	3000-6000	60	97	0.74 (0.477, 1.170)	0.934 (0.547, 1.593)	0.801
	>6000	47	37	1.535 (0.902, 2.613)	2.441 (1.197, 4.977)	0.014

Previous history of self-medication	Yes	169	91	24.123 (12.087, 48.223)	26.816 (13.064, 15,064)	0.001
	No	10	130	1		

**Table:** (b) Model 2: binary logistic regression analysis of factors associated with self-medication with conventional medicine

Characteristics		SMCM		COR with 95% CI	AOR with 95% CI	*p* value
		Yes	No			
Age group in years	18-27	19	174	0.620 (0.337, 1.139)	0.589 (0.311, 1.14)	0.103
	>28	31	176	1		

Monthly income						0.139
	<3000	25	134	2.425 (0.953, 6.170)	2.669 (1.013, 7.035)	0.047
	3000-6000	19	138	1.790 (0.686, 4.669)	2.154 (0.804, 5.771)	0.127
	>6000	6	78	1		

Previous history of self-medication	Yes	45	215	5.651 (2.188, 14.593)	5.223 (2.007, 13.592)	0.001
	No	5	135	1		

**Table:** (c) Model 3: binary logistic regression analysis of factors associated self-medication with herbal medicine

Characteristics		SMHM		COR with 95% CI	AOR with 95% CI	*p* value
		Yes	No			
Age group in years	18-27	63	130	0.643 (0.427, 0.966)	0.870 (0.534, 1.418)	0.576
	>28	89	118	1		

Monthly income						0.005
	<3000	60	99	1		
	3000-6000	49	108	0.749 (0.470, 1.193)	0.922 (0.543, 1.566)	0.763
	>6000	43	41	1.730 (1.014, 2.954)	2.739 (1.376, 5.452)	0.004

Previous history of self-medication	Yes	145	115	23.957 (10.781, 53.235)	27.164 (11.913, 61.935)	0.001
	No	7	133	1		

## Data Availability

All relevant data are available within the manuscript.

## Abbreviations

CM: Conventional medicine
ETB: Ethiopian birr
HM: Herbal medicine
UoGCSH: University of Gondar Comprehensive Specialized Hospital.

## Supplementary Materials

Supplementary 1 Supporting information.

Supplementary 2 Annex I: informed consent.

Supplementary 3 Annex II: a questionnaire.
