# Supplementary material for: Prevalence and Associated Factors of Self-Medication among Pregnant Women on Antenatal Care Follow-Up at University of Gondar Comprehensive Specialized Hospital in Gondar, Northwest Ethiopia: A Cross-Sectional Study
Source: Int J Reprod Med. 2020 Sep 29;2020:2936862. doi: 10.1155/2020/2936862 (PMC7545459; doi:10.1155/2020/2936862)
Supplement: Supplementary 1 — Supporting information. [file 2936862.f1.docx]

**Supporting information**

**Factors associated with self-medication**

Age (chi square value = 6.196; p value = 0.045), monthly income (chi square value = 6.992; p value = 0.030), marital status (chi square value = 8.210; p value = 0.042), and a previous history of self medication (chi square value = 9.382; p value = 0.003) showed a significant association with SMP. Distance from health facility, education level, religion, occupation, and place of residence didn’t show significant association with SMP (**Table 1**).

**Table 1: Cross-tabulation in Chi-Square Tests for factors associated with self-medication at UoGCSH, June 2019**

| Characteristics | | SMP | | Total | X^2^-value | p-value |
| --- | --- | --- | --- | --- | --- | --- |
|  |  | Yes | No |  |  |  |
| Marital status | Single | 3 | 9 | 12 | 8.210 | 0.043* |
|  | Married | 171 | 193 | 364 |  |  |
|  | Divorced or Widowed | 5 | 19 | 24 |  |  |
|  | Total | 179 | 221 | 400 |  |  |
| Occupation | Governmental employed | 44 | 81 | 125 | 9.598 | 0.093* |
|  | self-employee | 52 | 54 | 106 |  |  |
|  | Housewife | 63 | 63 | 126 |  |  |
|  | Farmer | 12 | 11 | 23 |  |  |
|  | Student | 2 | 7 | 9 |  |  |
|  | Unemployed | 6 | 5 | 11 |  |  |
|  | Total | 179 | 221 | 400 |  |  |
| Education level | Illiterate | 10 | 13 | 23 | 3.754 | 0.478* |
|  | Primary school (1-8) | 26 | 31 | 57 |  |  |
|  | Secondary school (9-12) | 73 | 78 | 151 |  |  |
|  | College/University student | 1 | 6 | 7 |  |  |
|  | Diploma/Degree | 69 | 93 | 162 |  |  |
|  | Total | 179 | 221 | 400 |  |  |
| Religion | Orthodox | 138 | 167 | 305 | 4.418 | 0.198* |
|  | Muslim | 37 | 43 | 80 |  |  |
|  | Protestant | 3 | 11 | 14 |  |  |
|  | Jehovah witness | 1 | 0 | 1 |  |  |
|  | Total | 179 | 221 | 400 |  |  |
| Place of residence | Urban | 152 | 185 | 337 | 0.108 | 0.778 |
|  | Rural | 27 | 36 | 63 |  |  |
|  | Total | 179 | 221 | 400 |  |  |
| Distance from a health facility | <5km | 100 | 137 | 237 | 1.755 | 0.416 |
|  | 5-10km | 48 | 54 | 102 |  |  |
|  | >10km | 31 | 30 | 61 |  |  |
|  | Total | 179 | 221 | 400 |  |  |
| Age group in year | 18-27 | 74 | 119 | 193 | 6.196 | 0.043 |
|  | >28 | 105 | 102 | 207 |  |  |
|  | Total | 179 | 221 | 400 |  |  |
| Monthly income | <3000 | 72 | 87 | 159 | 6.992 | 0.030 |
|  | 3000-6000 | 60 | 97 | 157 |  |  |
|  | >6000 | 47 | 37 | 84 |  |  |
|  | Total | 179 | 221 | 400 |  |  |
| Previous history of self medication( it is either by conventional or herbal medicine) | Yes | 169 | 91 | 260 | 123.205 | 0.0001 |
|  | No | 10 | 130 | 140 |  |  |
|  | Total | 179 | 221 | 400 |  |  |

*****Fisher exact test was used when the assumption was failed for Pearson Chi-Square.

**Factors associated with self-medication by using conventional medication**

The use of CM as a self medication showed a significant association with occupation (chi-square value = 13.692; p-value = 0.018) and a previous history of self medication (chi-square value = 24.463; p-value = 0.001). The remaining variables didn’t show a significant association with CM practices **(Table 2)**.

**Table 2: Cross-tabulation in Chi-Square Tests for factors associated with self-medication by using conventional medicationat UoGCSH, June 2019**

| Characteristics | | CMP | | Total | X^2^-value | p-value |
| --- | --- | --- | --- | --- | --- | --- |
|  |  | Yes | No |  |  |  |
| Marital status | Single | 1 | 11 | 12 | 1.947 | **0.810*** |
|  | Married | 48 | 316 | 364 |  |  |
|  | Divorced | 1 | 19 | 20 |  |  |
|  | Widowed | 0 | 4 | 4 |  |  |
|  | Total | 50 | 359 | 400 |  |  |
| Occupation | Gov't employed | 8 | 117 | 125 | 13.692 | **0.018*** |
|  | self-employee | 15 | 91 | 106 |  |  |
|  | Housewife | 18 | 108 | 126 |  |  |
|  | Farmer | 5 | 18 | 23 |  |  |
|  | Students | 0 | 9 | 9 |  |  |
|  | Unemployed | 4 | 7 | 11 |  |  |
|  | Total | 50 | 350 | 400 |  |  |
| Education level | Illiterate | 3 | 20 | 23 | 3.618 | **0.518*** |
|  | Primary school (1-8) | 7 | 50 | 57 |  |  |
|  | Secondary school (9-12) | 24 | 127 | 151 |  |  |
|  | College/University student | 0 | 7 | 7 |  |  |
|  | Diploma/Degree | 16 | 146 | 162 |  |  |
|  | Total | 50 | 350 | 400 |  |  |
| Religion | Christian | 41 | 264 | 305 | 9.705 | **0.077*** |
|  | Muslim | 8 | 72 | 80 |  |  |
|  | Protestant | 0 | 14 | 14 |  |  |
|  | Jehovah witness | 1 | 0 | 1 |  |  |
|  | Total | 50 | 350 | 400 |  |  |
| Place of residence | Urban | 39 | 298 | 337 | 1.682 | **0.195** |
|  | Rural | 11 | 52 | 63 |  |  |
|  | Total | 50 | 350 | 400 |  |  |
| Distance from a health facility | <5km | 24 | 213 | 237 | 3.013 | **0.222** |
|  | 5-10km | 16 | 86 | 102 |  |  |
|  | >10km | 10 | 51 | 61 |  |  |
|  | Total | 50 | 350 | 400 |  |  |
| Age in year | 18-27 | 19 | 174 | 193 | 2.404 | **0.13** |
|  | 28-37 | 31 | 176 | 207 |  |  |
|  | Total | 50 | 350 | 400 |  |  |
| Monthly income | <3000 | 25 | 134 | 159 | 3.737 | **0.154** |
|  | 3000-6000 | 19 | 138 | 157 |  |  |
|  | >6000 | 6 | 78 | 84 |  |  |
|  | Total | 50 | 350 | 400 |  |  |
| previous history of self medication | Yes | 45 | 215 | 260 | 15.69 | **0.001** |
|  | No | 5 | 135 | 140 |  |  |
|  | Total | 50 | 350 | 400 |  |  |

*****Fisher exact test was used when the assumption was failed for Pearson Chi-Square.

**Factors associated with self-medication by using herbal medicine**

There was evidence of association between age (chi-square value = 4.544; p-value = 0.039), monthly income (chi-square value = 9.280; p-value = 0.010), marital status (chi-square value = 7.783; p-value = 0.035) and previous history of self medication (chi-square value = 99.556; p-value = 0.001) with HM practice. However, the other variables didn’t show significant associations with HM practice (**Table 3)**.

**Table 3: Cross-tabulation in Chi-Square Tests for factors associated with self-medication by using herbal medicine**

**at UoGCSH, June 2019**

| Characteristics | | HMP | | Total | X^2^-value | p-value |
| --- | --- | --- | --- | --- | --- | --- |
|  |  | Yes | No |  |  |  |
| Marital status | Single | 2 | 10 | 12 | 7.783 | 0.035* |
|  | Married | 146 | 218 | 364 |  |  |
|  | Divorced | 3 | 17 | 20 |  |  |
|  | Widowed | 1 | 3 | 4 |  |  |
|  | Total | 152 | 248 | 400 |  |  |
| Occupation | Governmental employed | 39 | 86 | 125 | 6.890 | 0.208* |
|  | self-employee | 40 | 66 | 106 |  |  |
|  | Housewife | 54 | 72 | 126 |  |  |
|  | Farmer | 12 | 11 | 23 |  |  |
|  | Student | 2 | 7 | 9 |  |  |
|  | Unemployed | 5 | 6 | 11 |  |  |
|  | Total | 152 | 248 | 400 |  |  |
| Education level | Illiterate | 9 | 14 | 23 | 2.697 | 0.615* |
|  | Primary school (1-8) | 25 | 32 | 57 |  |  |
|  | Secondary school (9-12) | 58 | 93 | 151 |  |  |
|  | College/University | 1 | 6 | 6 |  |  |
|  | Student | 59 | 103 | 162 |  |  |
|  | Diploma/Degree | 152 | 148 | 400 |  |  |
|  | Total |  |  |  |  |  |
| Religion | Orthodox | 116 | 189 | 305 | 2.604 | 0.488* |
|  | Muslim | 33 | 47 | 80 |  |  |
|  | Protestant | 3 | 11 | 14 |  |  |
|  | Jehovah witness | 0 | 1 | 1 |  |  |
|  | Total | 152 | 248 | 400 |  |  |
| Place of Residence | Urban | 128 | 209 | 337 | 0.001 | 0.986 |
|  | Rural | 24 | 39 | 63 |  |  |
|  | Total | 152 | 248 | 400 |  |  |
| Distance from a health facility | <5km | 84 | 153 | 237 | 2.338 | 0.311 |
|  | 5-10km | 40 | 62 | 102 |  |  |
|  | >10km | 28 | 33 | 61 |  |  |
|  | Total | 152 | 248 | 400 |  |  |
| Age group in year | 18-27 | 63 | 130 | 193 | 4.544 | 0.039 |
|  | 28-37 | 89 | 118 | 207 |  |  |
|  | Total | 152 | 248 | 400 |  |  |
| Monthly income | <3000 | 60 | 99 | 159 | 9.280 | 0.010 |
|  | 3000-6000 | 49 | 108 | 157 |  |  |
|  | >6000 | 43 | 41 | 84 |  |  |
|  | Total | 152 | 248 | 400 |  |  |
| previous history of self medication | Yes | 145 | 115 | 260 | 99.556 | 0.001 |
|  | No | 7 | 133 | 140 |  |  |
|  | Total | 152 | 248 | 400 |  |  |

*****Fisher exact test was used when the assumption was failed for Pearson Chi-Square.
