# Supplementary material for: Prevalence and Associated Factors of Self-Medication among Pregnant Women on Antenatal Care Follow-Up at University of Gondar Comprehensive Specialized Hospital in Gondar, Northwest Ethiopia: A Cross-Sectional Study
Source: Int J Reprod Med. 2020 Sep 29;2020:2936862. doi: 10.1155/2020/2936862 (PMC7545459; doi:10.1155/2020/2936862)
Supplement: Supplementary 2 — Annex I: informed consent. [file 2936862.f2.docx]

## ANNEX I Informed consent

Greetings, this study aimed to assess the prevalence and factors associated with self-medication practice among pregnant women on antenatal care follow-up at university of Gondar Comprehensive Specialized Hospital in Gondar, Northwest Ethiopia. We would like you to participate in this study, politely.

There is no any medical or laboratory investigation for the purpose of this study. This study only collects data from patient interview. Any personal identifier will not be taken neither from patient nor medical record/chart. The collected data will be handled in secured manner in closed cabinet. The collected data will be analyzed by the investigators for the purpose of this study only.

The finding of this study will help to find and show any gap in case self-medication practice among pregnant women. After data collection investigators will try to fill any gap by giving education for the participants.

Any participant who hasn’t willingness or ready to participate can reject participation on this study. This questionnaire is voluntary and completely anonymous. If you choose not to participate your treatment will not be affected in any way**.** If you have any suggestions and concerns you can communicate with the investigators of this study; Mr Faisel Dula Sema ( B.pharm, MSC in clinical pharmacy ), Deres Gezahegn Addis (B.pharm), Eshetie Azezew Melese (B.pharm), Demeke Dana Nassa (B.pharm), and Zemene Demelash Kifle (B.pharm MSC in pharmacology).

**Complimentary information of the investigators**:

**Faisel Dula** **Sema** (Department of Clinical Pharmacy, School of Pharmacy, College of Medicine and Health Sciences, University of Gondar, Ethiopia )

**Email;** Faiselye1203@gmail.com  **Phone number; +251912805424**

**Zemene Demelash Kifle** (Department of pharmacology, School of Pharmacy, College of Medicine and Health Sciences, University of Gondar, Ethiopia)

**Email;** [zemene2010@gmail.com](mailto:zemene2010@gmail.com) **Phone number፣ +251918026724**

Deres Gezahegn Addis (Schools of Pharmacy, College of Medicine and Health Sciences, University of Gondar, Ethiopia)

**Email;** [deresgezahegn121@gmail.com](mailto:deresgezahegn121@gmail.com) **Phone number፣ +251928337535**

Eshetie Azezew Melese (Schools of Pharmacy, College of Medicine and Health Sciences, University of Gondar, Ethiopia)

**Email;** [eshetieazezew2011@gmail.com](mailto:eshetieazezew2011@gmail.com) **Phone number፣ +251918308732**

Demeke Dana Nassa (Schools of Pharmacy, College of Medicine and Health Sciences, University of Gondar, Ethiopia)

**Email;** [demeked24@gmail.com](mailto:demeked24@gmail.com)

We would like you to show your willingness and permission to participate in this study.

In the manner that without personal identifier I declare that any data that need for this research can be used and I am ready to participate

**Full name date signature**

**------------------------------- -------------------------- ------------------------------**

**የፈቃደኝነት መግለጫ**

ጤና ይስጥልኝ፤ በ ጎንደር ዩኒቨርስቲ አጠቃላይ ስፔሻላይዝድ ሆስፒታል ለክትትል በመጡ ነብሰጡር እናቶች ላይ ሃኪም ያላዘዘውን መድሃኒት መውሰድን በተመለከተ በጎንደር ዩኒቨርሲቲ ሆስፒታል ጥናት በማካሄድ ላይ እንገኛለን። እርሶም በ ዚ ጥናት ላይ እንዲሳተፉ በ ትህትና እንጠይቃለን።

ለዚህ ጥናት ሲባል የሚከናወን ምንም አይነት ሕክምናም ሆነ የ ላቡራቶሪ ምርመራ አይኖርም። ለ ጥናቱ የሚያስፈልጉ መረጃዎች በዚህ ምርምር ከሚሳትፉ ታካዎች በቃለ መጠይቅ ሚሰበሰቡ ይሆናል። ሁሉም መረጃዎችህ ለያንዳንዱ ተሳታፊ በተሰጠ ሚስጥራዊ ቁጥር የሚሰበሰቡ ይሆናል። በ ዚህም ጊዜ ምንም አይነት የታካሚውን ማንነት ሊገልጹ የሚችሉ መረጃዎች ከታካሚውም ይሁንከ ከታካሚውም የ ህክምናው ሰነድ አይወሰድም። ነገር ግን ከታካሚው የተሰበሰበው መረጃ ተሳታፊዎችን መለየት በማይቻልበት መልኩ ለእያንዳንዱ በተሰጡ ሚስጥራዊ ቁጥር ስር ተሰብስበው በተማራማሪዎቹ የሚተነተኑ ይሆናል። ተሰብስቦ የተተነተነው መረጃ ለዚ ምርምር ጥቅም ላይ ይውላል።

የዚህ ጥናት ጥናት ግኝት ነብሰጡር እናቶች ላይ ሃኪም ያላዘዘውን መድሃኒት መውሰድን በተመለከተ ያለውን ክፍተት ለማወቅ እና ለማሳየት ይጠቅማል። ጥናቱ ከተጠናቀቀ ብኋላ የጥናቱ ውጤት ለታካሚዎቹ ይገለፃል።

ማንኛውም ታካሚ በዚህ ጥናት ለመሳትፍ ፈቃደኛ ካልሆነ አለመሳተፍ ይችላል። ይሄ መጠይቅ የሚሞላው በፈቃደኝነት ላይ ተመስርቶ ብቻ ነው።ለመሳትፍ ፈቃደኛ ያልሆነ ታካሚ በምንም አይነት መልኩ የሚያገኝውን ህክምና ወይም ማንኝውም አገልግሎት አይስተጋጎልም።

ተጨማሪ ማንኛውም ሃሳብ እና አስተያየት ካሎት የዚህ ጥናት ተመራማሪ የሆኑትን አቶ ፈይሰል ዱላ (ቢ ኤስ ሲ በ ፋርማሲ፣ ኤም ኤስ ሲ በ ክሊኒካል ፋርማሲ ) ፣ አቶ ዘመነ ደመላሽ ክፍሌ (ቢ ኤስ ሲ በ ፋርማሲ፣ ኤም ኤስ ሲ በፋርማኮሎጂ) ፣ አቶ ደረስ ገዛኸኝ አዲስ (ቢ ኤስ ሲ በ ፋርማሲ )፣ አቶ እሸቴ አዘዘው መለሰ (ፋርማሲ(ቢ ኤስ ሲ በ ፋርማሲ) እና አቶ ደመቀ ዳና ናሳ (ቢ ኤስ ሲ በ ፋርማሲ) ።

**ስለ ተመራማሪዎቹ ተጨማሪ መረጃ**

**ፈይሰል ዱላ** (የክሊኒካል ፋርማሲ ት/ቤት ፣ የ ፋርማሲ ት/ቤት ፣ ህክምና ጤና ሳይንስ ኮሌጅ ፣ ጎንደር ዩኒቨርስቲ ፣ጎንደር ፣ ኢትዮጲያ)

**ኢ-ሜይል**፡ Faiselye1203@gmail.com **ስልክ ቁጥር ፣ +251912805424**

ዘመነ ደመላሽ ክፍሌ ((የፋርማኮሎጂ ት/ቤት ፣ የ ፋርማሲ ት/ቤት ፣ ህክምና ጤና ሳይንስ ኮሌጅ ፣ ጎንደር ዩኒቨርስቲ ፣ጎንደር ፣ ኢትዮጲያ)

**ኢ-ሜይል**፡ [zemene2010@gmail.com](mailto:zemene2010@gmail.com) **ስልክ ቁጥር ፣ +251918026724**

አቶ ደረስ ገዛኸኝ አዲስ (ፋርማሲ ት/ቤት፣ ጎንደር ዩኒቨርስቲ ህክምና ጤና ሳይንስ ኮሌጅ፣ ጎንደር ዩኒቨርሲቲ፣ ኢትዮጲያ )

**ኢ-ሜይል**፡ [deresgezahegn121@gmail.com](mailto:deresgezahegn121@gmail.com) **ስልክ ቁጥር ፣ +251928337535**

አቶ እሸቴ አዘዘው መለሰ (ፋርማሲ ት/ቤት፣ ጎንደር ዩኒቨርስቲ ህክምና ጤና ሳይንስ ኮሌጅ፣ ጎንደር ዩኒቨርሲቲ፣ ኢትዮጲያ )

**ኢ-ሜይል**፡ [eshetieazezew2011@gmail.com](mailto:eshetieazezew2011@gmail.com) **ስልክ ቁጥር ፣ +251918308732**

አቶ ደመቀ ዳና ናሳ (ፋርማሲ ት/ቤት፣ ጎንደር ዩኒቨርስቲ ህክምና ጤና ሳይንስ ኮሌጅ፣ ጎንደር ዩኒቨርሲቲ፣ ኢትዮጲያ )

**ኢ-ሜይል**፡ [demeked24@gmail.com](mailto:demeked24@gmail.com)

በ ዚህ ጥናት ውስጥ ለመሳተፍ ለመሳተፍ ፈቃደኛ መሆንዎትን መስማማትዎን እንዲገልፁልን በትህትና እንጠይቃለን። በዚህ ጥናት ውስጥ የኔን ማንነት በማይገልጥ መልኩ ሁሉን የሚያስፈልግ መረጃ ለጥናቱ እንዲውልና በዚህ ጥናት ለመሳተፍ ፈቃደኛ መሆኔን አሳውቃልው።

**ሙሉ ስም ቀን ፊርማ**

----------------------------- ----------------------- ----------------------
