# Supplementary material for: Prevalence and Associated Factors of Self-Medication among Pregnant Women on Antenatal Care Follow-Up at University of Gondar Comprehensive Specialized Hospital in Gondar, Northwest Ethiopia: A Cross-Sectional Study
Source: Int J Reprod Med. 2020 Sep 29;2020:2936862. doi: 10.1155/2020/2936862 (PMC7545459; doi:10.1155/2020/2936862)
Supplement: Supplementary 3 — Annex II: a questionnaire. [file 2936862.f3.docx]

# ANNEX II A Questioner

This questioner is prepared to assess the prevalence and associated factors of self-medication practice among pregnant women on antenatal care follow-up at university of Gondar Comprehensive Specialized Hospital.

**Section-1: Socio-Demographic Information**

1. Age (in years) _______________

2. Marital status

a) Single b) Married

c) Divorced d) Widowed

3. Occupation

a) Governmental employed b) self-employee

c) Housewife d) Farmer

e) Student f) others (specify) ________________

4. Monthly income (in ETB) ________________________________________

5. Education level

a) Illiterate b) Primary school (1-8)

c) Secondary school (9-12) d) College/University student

e) Diploma/Degree

6. Religion

a) Orthodox b) Muslim

c) Protestant d) Others ______________

7. Place of Residence

a) Urban b) Rural

8. Distance from health facility (hospital or health center)

a) In km ______________

b) In time ______________

**Section-2: Obstetrics Information**

9, Number of gravida ____________________________

10, Number of parity ____________________________

11. Number of child _____________________________

12. History of abortion

a. No b. Yes

13. If yes, the reason for abortion is

a, health problem b, low economic level

c. unwanted pregnancy d. other

14. Stage of pregnancy

a) First trimester b) Second trimester c) Third trimester

**Section-3: Pregnant women’s attitude on self-medication**

15. Do you believe that self-medication is important for maternal health?

a, Yes b, No c, I have no any idea

16. Do you believe that self-medication is important for fetal health?

a, Yes b, No c, I have no any idea

17. At which trimester of pregnancy the negative effect of self-medication can mostly occur?

a, First trimester b, Second trimester

c, Third trimester d, I have no any idea

18. Do you believe that unusual health problem can occur on the infant(s) due to self-medication during pregnancy?

a, Yes b, No c, I have no any idea

**Section-4: Self-Medication Practice by conventional medicine**

19. Do you have history of self-medication by conventional medicine (modern medicine)?

a) YES b) NO

20. Have you practiced self-medication by conventional medicine (modern medicine) during the current pregnancy?

a) YES b) NO

**(If your answer is “NO” for question 20, please jump to Section 5 Q-27 )**

21. What makes you to practice self-medication by conventional medicine (modern medicine) (Reason for selecting self-medication) during the current pregnancy?

a) Time saving

b) Easily available

c) Better knowledge about the disease and the treatment

d) Had prior experience to the drug

e) Other reason (specify) _______________________________

22. For what types of ailments have you practiced self-medication by conventional medicine (modern medicine) during the current pregnancy?

a) Headache b) Nausea/Vomiting

c) Typhoid d) UTI

e) Common cold f) Diarrhea

g) Cough h) Other (specify) ______________________________

23. What is the name of the medicine(s) that you have used for self-medication by conventional medicine (modern medicine) during the current pregnancy?

a) Paracetamol b) Aspirin

c) Tetracycline d) amoxicillin

e) Cough syrup f) Hyoscine

g) Amoxicillin h) I don’t remember i) other (specify) __________

24. Who is your source of information about the conventional or modern medicine(s)?

a) Yourself b) Your husband

c) Your friend d) your neighbor

e) Internet f) Pharmacist/Druggist

g) Other health professional h) other (specify) __________________________

25. From where did you get the conventional or modern medicine(s) for self-medication?

a) Neighbors b) Friends c) Shops

d) Private drug sellers (Community pharmacy and drug stores)

e) Other (specify) ____________________________________

26. What did you know about the conventional or modern medicine(s) that you have used for self-medication?

a) Dose b) Side effects

c) How to take d) No information

**Section-5: Herbal Medicine Use Practice**

27. Do you have history of self-medication practice by herbal medicine?

a) YES b) NO

28. Have you practiced herbal medicine for self- medication during the current pregnancy?

a) YES b) NO

**(If your answer for Q-28 is “No” you have finished)**

29. What makes you to practice herbal medicine use (Reason for selecting herbal medicine use) for self-medication during the current pregnancy?

a) Herbal medicines are effective than conventional medicines

b) Herbal medicines have fewer side effects

c) Herbal medicines have lower cost

d) Herbal medicines are accessible without prescription

e) Herbal medicines are the only option

e) Other (specify) _______________________________

30. For what purpose and ailments have you used herbal medicine for self medication?

a) Headache b) Nausea/Vomiting

c) Typhoid d) UTI

e) Common cold f) Diarrhea

g) To facilitate labor h) to prevent abortion

i) Other (specify) _____________________________

31. What Types of herb(s) have you used for self medication?

a) Ginger b) Garlic

c) Ruta chalepensis (Tena Adam) d) Ocimum lamifolium (Damakese)

e) Thyme (Tosign) f) other (specify) _________________

32. Who is your source of information about herbal medicine for self medication?

a) Traditional healers b) Health professionals

c) Religious leaders d) Family and friends

e) Neighbors f) other (specify) _____________________________

33. From where did you get the herbal medicine for self medication?

a) Self-preparation b) Traditional healers/Herbalist

c) Traditional birth attendants d) Market place

e) Neighbors f) other (specify) __________________________

***Thank you for your willingness to participate in the study!!***

*Name of data collector_______________________________*

*Signature_________________________________________*

*Date of data collection________________________________*

*Name of health facility_________________________________________*

**አማርኛ መጠይቅ**

**መጠይቅ፥** ይህ መጠይቅ በ ጎንደር ዩኒቨርስቲ አጠቃላይ ስፔሻላይዝድ ሆስፒታል ለክትትል በመጡ ነብሰጡር እናቶች ላይ ሃኪም ያላዘዘውን መድሃኒት መውሰድን በተመለከተ ስርጭቱን እና ምክንያቶቹን ለማጥናት የተዘጋጀ ነው።

**ክፍል-1:- ማህበራዊ-ግላዊ መረጃዎች**

1. ዕድሜ (በዓመት) _______________

2. የጋብቻሁኔታ

ሀ) ያላገባች

ለ) ያገባች

ሐ) የተፋታች

መ) የሞተባት

3. ሥራ

ሀ) የመንግስት ሰራተኛ

ለ) በግሉ የሚሠራ

ሐ) የቤት እመቤት

መ) ገበሬ

ሠ) ተማሪ

ረ) ሌሎች (ይግለጹ) ________________

4. ወርሃዊገቢ (በብር) ________________

5. የትምህርትደረጃ

ሀ) ያልተማረች

ለ) የመጀመሪያ ደረጃ ትምህርት የተማረች (1-8)

ሐ) ሁለተኛ ደረጃ ትምህርት የተማረች (9-12).

መ) ኮሌጅ / ዩኒቨርሲቲ የተማረች

ሠ) ዲፕሎማ / ዲግሪ ያላት

6. ሃይማኖት

ሀ) ኦርቶዶክስ)

ለ) ሙስሊም

ሐ) ፕሮቴስታንት

መ) ሌሎች ______________

7. የመኖሪያቦታ

ሀ) ከተማ ለ) ገጠር

8. ከጤናተቋማት (ሆስፒታልወይምጤናማዕከል)

ሀ) <5 ኪሜ ለ) 5-10 ኪሎሜትር ሐ) > 10 ኪሜ

**ክፍል-2:- የርግዝና መረጃ**

9. ስንተኛ እርግዝናዎ ነው? ____________________________

10. ስንት ጊዜ ወልደዋል? ____________________________

11. የልጆችዎ ቁጥር ስንት ነው? ______________________

12. ከዚህ በፊት ውርጃ አጋጥሞዎት ያውቃል? ____________________

ሀ. አያውቅም ለ. አዎ

13. መልሰዎ አዎ ከሆነ፣ ፅንሱ የወረደበት ምክንያት ምንድን ነበር? ____________________

14. የእርግዝና ወቅት ወይም ወሰን (ወር)፥ እርግዝናዎ ስንት ጊዜ ሆነው? በወር ________________ በሳምንት _______________

ሀ) የመጀመሪያ ወሰን (1 – 3 ወር) ለ) ሁለተኛ ወሰን (3 – 6 ወር)

ሐ) ሶስተኛረጃ ወሰን (6 – 9 ወር)

**ክልፍ3፦ያለሃኪም ትእዛዝ ስለሚወሰዱ መዲሃኒቾች የነፍሰጡር እናቶች ግንዛቤ**

15. በሃኪም ያልታዘዙ መድሃኒቶች ለእናቶች ጤና አስፈላጊ ነው ብለው ያሚናሉ?

A, አዎ B, አላምንም

16. በሃኪም ያልታዘዙ መድሃኒቶች ለጽንሱ ጤንነት አስፈላጊ ናቸው ብለው ያሚናሉ?

A, አዎ B, አላምንም C, አላውቅም

17. በሃኪም ያልታዘዙ መድሐኒቶችን መጠቀም ጉዳቱ በየትኛው የእርግዝናወቅት (ወር) በአብዛኛው ሊከሰት ይችላል?

A, 1-3 ወር B, 4-6

C, 7-9 D, አላውቅም

18. በሃኪም ያልታዘዙ መድሃኒቶችን በመውሰድ ምክንያት ያልተለመደ የጤና እክል ያለባችው ህጻናት ሊወለዱ ይችላሉ?

A, አዎ B, አይችሉ C, አላውቅም

**ክፍል -4:- ያለሃኪም ትእዛዝ ዘመናዊ መዲሃኒት ራስን ለማከም የመጠቀም ልምድ**

19. ከዚህ በፊት ያለሃኪም ትእዛዝ ዘመናዊ መድሃኒት ወስደው ያውቃሉ?

ሀ) አዎ ለ) አላውቅም

20. በአሁኑ የእርግዝና ወቅት ያለሃኪም ትእዛዝ ዘመናዊ መድሃኒት ወስደዋል?

ሀ) አዎ ለ) አልወሰድኩም

**(በቁጥር 3 ውስጥ የእርስዎ መልስ አልወሰድኩም ከሆነ ወደ ክፍል-5 ጥያቄ 27 ይለፉ)**

21. በጥያቄ ቁጥር 20 ውስጥ የእርስዎ መልስአዎ ከሆነ፣ ያለሃኪም ትእዛዝ መዲሃኒት ለመውሰድ ያስገደደዎት ምክንያት ምንድነው?

ሀ) ጊዜን ለመቆጠብ

ለ) በቀላሉ ስለሚገኝ

ሐ) ስለ በሽታው እና ህክምናው በቂዕውቀት ስላለኝ

መ) ለስለመዲሃኒቱ በፊት የነበረ ልምድ ስላለኝ

ሠ) ሌላምክንያትከሆነ (ዝርዝሩንይግለጹ) _______________________________

22. ያለሃኪም ትእዛዝ መዲሃኒት የወሰዱት ለምን አይነት ህመሞች ነው?

ሀ) የራስ ምታት ለ) ማቅለሽለሽ / ማስታወክ

ሐ) ታይፎይድ መ) የሽንት ቱቦዎች መቁሰል

ሠ) ጉንፋን ረ) ተቅማጥ

ሰ) ሳል ሽ) ሌላ (ይግለጹ) __________

23. ያለሃኪም ትእዛዝ የወሰዱት መድሃኒት (ቶች) ስም ምንድን ነው?

ሀ) ፓራስታሞል (Paracetamol)

ለ) አስፕሪን (Aspirin

ሐ) ቴትራሳይክሊን (Tetracycline)

መ) አሞክሲሲሊን (amoxicillin)

ሠ) የሳልሲረፕ (Cough syrup)

ረ) ሃዮሲን (Hyoscine)

ሰ) ሚዝል ወይም ሜትሮኒዳዞል (Metronidazol)

ሸ)አላስታውስውም

ቀ) ሌላ (ይግለጹ)________________

24. ስለመድሃኒቱ የመረጃ ምንጮችዎ ማን (እነማን) ናቸው?

ሀ) እራስዎ

ለ) ባለቤትዎ

ሐ) ጓደኛዎ

መ)ጎረቤትዎ

ሠ) ኢንተርኔት

ረ) የመድሃኒትባለሙያ

ሰ) ሌላየጤናባለሙያ

ሸ) ሌላ (ይግለጹ) ________

25. መድሃኒቶቱን ያገኙት ከየት ነው?

ሀ) ከጎረቤቶች

ለ) ከጓደኞች

ሐ) ከሱቆች

መ) የግል መዲሃኒት ሻጮች (በግል መድኃኒት ቤት እና መድኃኒት መደብሮች)

ሠ) ሌላ (ይግለጹ)___________________

26. ያለሃኪም ትእዛዝ ስለተጠቀሟቸው መድሃኒቶች ምን ያውቃሉ?

ሀ) መጠን

ለ) የጎንዮሽ ጉዳቶችን

ሐ) እንዴት እንደሚዎሰድ

መ) ምንም መረጃ የለም

**ክፍል -5፡ያለሃኪም ትእዛዝ የባህላዊ መዲሃኒት ራስን ለማከም የመጠቀም ልምድ**

27. ከዚህ በፊት ያለሃኪም ትእዛዝ ከዕፅዋት የተቀመሙ የባህል መድሃኒት ወስደው ያውቃሉ?

ሀ) አዎ ለ) አይደለም

28. በአሁኑ ወቅት እርግዝና ጊዜ ከዕፅዋት የተቀመሙ የባህል መድኃኒት ይጠቀማሉ?

ሀ) አዎ

ለ) አልጠቀምም

**በቁጥር 28 ላይ ያለው ጥያቄ መልሶ አልጠቀምም ከሆነ እዚህ ጨርሰዋል**

29. በቁጥር 3 ውስጥ የእርስዎ መልስ አዎ ከሆነ፣ከዕፅዋት የተቀመሙ የባህል መድሃኒቶችን እንዲጠቀሙ ያደረገዎትምክንያትምንድንነው?

ሀ) ከእጽዋት የሚቀመሙ መድሃኒቶች ከዘመናዊ መድሃኒቶች የበለጠውጤታማ ስለሆኑ

ለ) ከዕፅዋት የተቀመሙ መድኃኒቶች የጎንዮሽ ጉዳታቸው አነስተኛ ስለሆነ

ሐ) ከዕፅዋት የሚገኙ መድሃኒቶች አነስተኛ ዋጋ ስላላቸው

መ) ከዕፅዋት የሚቀመሙ መድኃኒቶች ያለማዘዣ ስለሚገኙ

ሠ / ሌላ (ይግለጹ) _______________________________

30. ከዕፅዋት የተቀመሙ የባህል መድሃኒቶችን ለምን አይነት ህመም ህክምና ይጠቀማሉ?

ሀ) የራስምታት

ለ) ማቅለሽለሽ / ማስታወክ

ሐ) ታይፎይድ

መ) የሽንት ቱቦ ኢንፌክሽን

ሠ) ጉንፋን

ረ) ተቅማጥ

ሰ) ምጥን ለማፋጠን

ሸ) ውርጃን ለመከላከል

ቀ) ሌላ (ይግለጹ)_____________

31. የትኞቹን ኣይነት ከዕፅዋት የተቀመሙ የባህል መድሃኒቶች ነው የሚጠቀሙት?

ሀ) ዝንጂብል

ለ) ነጭ ሽንኩርት

ሐ) ጢናዓዳም

መ) ዳማከሴ

ሠ) ጦስኝ

ረ) ሌላ (ዝርዝር ይግለጹ) ___________

32. ስለ ከዕጽዋት የሚቀመሙ የባህል መድኀኒቶች መረጃ የሚያገኙት ከማን ነው?

ሀ) የባህላዊ መዲሃኒት ቀማሚዎች

ለ) የጤና ባለሙያዎች

ሐ) የሃይማኖት መሪዎች (አባቶች)

መ ) ቤተሰብ እና ጓደኞች

ሠ) ጎረቤት

ረ) ሌላ (ይግለጹ) ________________

33 ከዕጽዋት የሚቀመሙ የባህል መድኀኒቶች ከየትያገኛሉ?

ሀ) እራሴ እያዘጋጀሁ

ለ) ከባህላዊ ሐኪሞች (ዕጽዋት ቀማሚዎች)

ሐ) ከባህላዊ አዋላጆች

መ) ከገበያ ቦታ

ሠ) ከጎረቤት

ረ) ሌላ (ይግለጹ)____________

***በጥናቱ ለመሳተፍ ፈቃደኛ በመሆነዎ ከልብ እናመሰግናለን !!***

*የመረጃ ሰብሳቢው ስም _______________________________*

*ፊርማ _________________________________________________*

*መረጃው የተሰበሰበበት ቀን ________________________________*

*የጤና ተቋሙ ስም ________________________________________*
